# Real world long-term impact of intensive treatment on disease activity, disability and health-related quality of life in rheumatoid arthritis

**DOI:** 10.1186/s41927-019-0054-y

**Published:** 2019-02-25

**Authors:** Nicola J. Gullick, Fowzia Ibrahim, Ian C. Scott, Alexandra Vincent, Andrew P. Cope, Toby Garrood, Gabriel S. Panayi, David L. Scott, Bruce W. Kirkham, On behalf of TITRATE Programme Investigators, Heidi Lempp, Jackie Sturt, Sofia Georgopoulou, Louise Prothero, Naomi Martin, Richard Jenner, Isabel Neatrour, Rhiannon Baggott, Fowzia Ibrahim, Brian Tom, Allan Wailoo, Jonathan Tosh, James Galloway, Gabrielle Kingsley, David Scott, Brian Tom, Fowzia Ibrahim, Yujie Zhong, Aneela Mian, James Galloway, David L. Scott

**Affiliations:** 1 Department of Rheumatology, University Hospitals Coventry and Warwickshire NHS Trust, Coventry, UK; 2 Department of Rheumatology, 3rd Floor, Weston Education Centre, King’s College London, Cutcombe Road, London, UK; 3 Research Institute for Primary Care & Health Sciences, Primary Care Sciences, Keele University, Keele, Staffordshire UK; 4 Department of Rheumatology, Haywood Hospital, High Lane, Burslem, Staffordshire UK; 5 Department of Rheumatology, Guy’s and St Thomas’ NHS Trust, 4th Floor, Tower Wing, Guy’s Hospital, Great Maze Pond, London, UK; 6 Academic Department of Rheumatology, Centre for Molecular and Cellular Biology of Inflammation, 1st Floor, New Hunt’s House, Guy’s Campus, King’s College London, Great Maze Pond, London, UK

**Keywords:** Temporal RA change, Intensive treatment, Rheumatoid arthritis

## Abstract

**Background:**

The emphasis on treating rheumatoid arthritis (RA) intensively reduces disease activity but its impact in routine care is uncertain. We evaluated temporal changes in disease activities and outcomes in a 10-year prospective observational cohort study of patients in routine care at one unit.

**Methods:**

The Guy’s and St Thomas’ RA cohort was established in 2005. It involved most RA patients managed in this hospital. Clinical diagnoses of RA were made by rheumatologists. Patients were seen regularly in routine care. Each visit included measurement of disease activity scores for 28 joints (DAS28), health assessment questionnaire scores (HAQ) and EuroQol scores. Patients received intensive treatments targeting DAS28 remission.

**Results:**

In 1693 RA patients mean DAS28 scores fell from 2005 to 15 by 11% from 4.08 (95% CI: 3.91, 4.25) in 2005 to 3.64 (3.34, 3.78); these falls were highly significant (*p* < 0.001). DAS28 components: swollen joint counts fell by 32% and ESR by 24%; in contrast tender joint counts and patient global assessments showed minimal or no reductions. The reduction in DAS28 scores was predominantly between 2005 and 2010, with no falls from 2011 onwards. Associated with falls in mean DAS28s, patients achieving remission increased (18% in 2005; 27% in 2015) and the number with active disease (DAS28 > 5.1) decreased (25% in 2005; 16% in 2015). In 752 patients seen at least annually for 3 years, persisting remission (68 patients) and intermittent remission (376 patients) were associated with less disability and better health related quality of life. Over time biologic use increased, but they were used infrequently in patients in persistent remission.

**Conclusions:**

Over 10 years an intensive management strategy in a routine practice setting increased combination DMARD and biologic use: disease activity levels declined; this association is in keeping with a causal relationship. Patients who achieved remission, even transiently, had better functional outcomes than patients never achieving remission.

**Electronic supplementary material:**

The online version of this article (10.1186/s41927-019-0054-y) contains supplementary material, which is available to authorized users.

## Background

In recent years the management of rheumatoid arthritis (RA) has been transformed. Methotrexate is used earlier, access to biologics has increased, and effective combinations of conventional disease modifying anti-rheumatic drugs (DMARDs) have been identified. National and international guidelines highlight the value of using these approaches in early, intensive and effective treatment [1–4]. The treat to target initiative has provided additional support [5, 6]. The overall impact of these innovations on patient outcomes in routine care settings remains uncertain.

Over 30 years ago, Silman and colleagues [7] suggested RA severity was declining. Many groups subsequently reported temporal improvements in RA. Some focussed on disease activity in early RA [8–12]; others concentrated on disease activity in established RA [13–17]. A few assessed erosive damage [18, 19] and joint replacement surgery [20, 21]. All studies provide some evidence of temporal improvements in RA outcomes. Such improvements could reflect treatment innovations and implementation of guidelines recommendations. Other potential influences include earlier referral resulting in more patients receiving effective therapy during the initial “window of opportunity” [22], changes in the clinical phenotype of RA [23] and changes in the relationship of RA to comorbid conditions [24].

More information is needed on the benefits of implementing intensive treatment strategies in routine clinics and the potential for further improvements. We examined both questions in a single-centre prospective observational study which recorded changes in disease activity, disability and health related quality of life (HRQoL) in patients managed intensively between 2005 and 2015.

## Methods

### Patients

Patients attending the Guy’s and St Thomas’ NHS Trust RA Centre formed a prospective longitudinal observational cohort study [25]. The majority of RA patients managed at this hospital were included. All patients had clinical diagnoses of RA made by experienced rheumatologists. They were seen regularly for routine care and each visit involved a clinical review and assessment of key clinical outcomes. Management followed the treat to target approach with an aim of reaching DAS28 remission defined as DAS28 < 2.6 [5, 25]. The patients were analysed in two ways: firstly, all patients in whom data was available; secondly, patients who were followed for three years or more. Details were collected about age, disease duration, sex and ethnicity.

### Treatments

Patients received treatment with DMARDs and biologics in line with existing English guidance about these treatments using a goal-directed strategy [26]. They received intensive DMARDs, often given in combination, and also had biologics when they met the existing guidelines from the National Institute for Health and Care Excellence (NICE). These English guidelines have changed over time and the approach taken reflected the guidance existing at the time treatment decisions were made and guidance from EULAR about treat to target [5, 27].

### Outcomes

The disease activity score 28 (DAS28), incorporating swollen and tender joint counts, patient’s global assessments, and erythrocyte sedimentation rates (ESR) evaluated patients’ current status [28], and was used to guide treatment decisions. The Heath Assessment Questionnaire (HAQ) measured disability [29]. The EuroQol 5-dimension scale (EQ5D-3 L) measures health-related quality of life [30]; it can be used to estimate health utility and has been widely used in RA. Flares were also assessed as changes between DAS28 assessments in which there was an increase in score of 0.60 or more [31].

### Data collection

Data were captured in the electronic healthcare records for inflammatory arthritis patients attending outpatient clinics at Guy’s and St Thomas’ Hospital NHS Foundation Trust (GSST). These records are within the Trust’s Electronic Patient Records system which provides laboratory results and patient demographics. Clinical data on a patient’s treatment, disease activity, disability level, and health-related quality of life (HRQoL) were routinely captured at their clinic appointment and were used for patients’ routine care.

### Analyses

Data management and analyses used Stata (version 14.0, StataCorp, College Station, TX). Descriptive analyses used numbers of patients and percentages and mean scores with standard deviations or 95% confidence intervals (CI). We used mixed models to examine the changes in DAS28 and its components over time. We also used trend analysis to take into account repeated measures from the same patient. Subgroups were compared by Chi-Squared analyses or by one-way analysis of variance.

### Ethics approval

Ethics approval for analysis of this routine clinical data was obtained from the Health Research Authority (IRAS project ID**:** 209418).

## Results

### Patients studied

#### All patients

Between 2005 and 2015 1693 RA patients were entered into the database. Most were female (1262, 75%) and Caucasian (1134, 67%). Their mean age was 55 years and mean disease duration was 11 years. The median time between assessments was 3 (IQR: 2–6) months and the mean was 5.3 (SD 6.6) months. Details of the patients are shown in Table 1. Numbers of patients with data in each calendar year are shown in Additional file 1: Table S1. Out of the total sample 337/1693 (19%) had at least one missing DAS28 during follow up. Similar proportions of HAQ and EQ5D data were missing (22 and 20% respectively).

**Table 1:** Baseline Characteristics Of Patients Studied: Number (%) or Mean (SD) Values Are Shown

		All Patients	Patients Followed Over Three Or More Years	Patients Followed Less Than Three Years
		*(n = 1693)*	*(n = 752)*	*(n = 941)*
Gender, n (%)	*Female*	1262 (75%)	579 (77%)	683 (73%)
	*Male*	412 (24%)	165 (22%)	247 (26%)
	*Missing*	19 (1%)	8 (1%)	11 (1%)
Ethnicity, n (%)	*Caucasian*	1134 (67%)	540 (72%)	594 (63%)
	*Black African/Caribbean*	209 (12%)	100 (13%)	109 (11%)
	*Asian/other background*	128 (8%)	52 (7%)	76 (8%)
	*Not Stated*	79 (5%)	27 (4%)	52 (6%)
	*Missing*	143 (8%)	33 (4%)	110 (12%)
Age (years)		55 (16)	55 (15)	55 (14.7)
Disease Duration (years)		11 (10)	10 (10)	10 (9.7)
Tender Joint Counts (28 joints)		5.0 (6.6)	4.8 (6.0)	5.2 (6.5)
Swollen joint Counts (28 joints)		2.9 (4.7)	3.2 (3.9)	3.3 (4.3)
Erythrocyte Sedimentation Rate (mm/hr)		22 (21)	22 (21)	21.5 (22)
Patient Global Assessment (mm)		46 (28)	44 (27)	45 (28)
DAS28		3.83 (1.63)	3.79 (1.50)	3.85 (1.62)
Health Assessment Questionnaire		1.18 (0.83)	1.22 (0.86)	1.18 (0.83)
EQ5D-3 L		0.53 (0.34)	0.52 (0.33)	0.53 (0.34)

#### Patients followed over three or more years

Seven hundred fifty-two patients were seen at least annually over three years or more. Their baseline features were similar to those of the overall group (Table 1). The median time between assessments was 3.5 (IQR 1–5) months and mean was 5.0 (SD: 5.5) months.

### Changes in DAS28

#### All patients

**Fig. 1:**
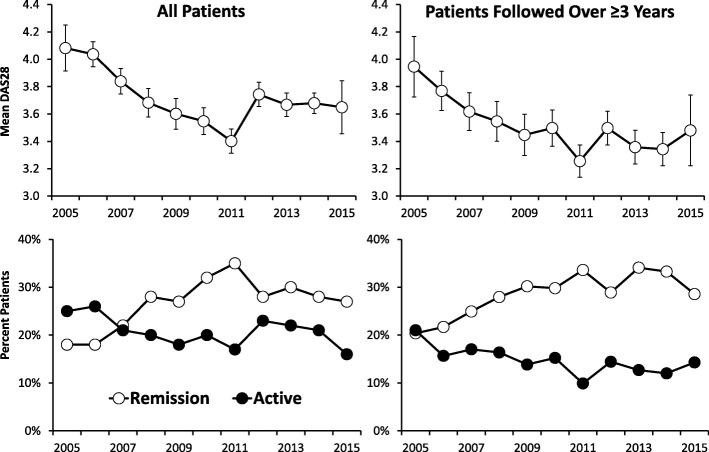
Changes in DAS28 (Means with 95% Confidence Intervals) and Percent Patients in Remission and with Active Disease

There were 10,773 measures of DAS28 in the 1693 patients. DAS28 scores fell when assessed as mean changes by calendar years (Fig. 1). The mean DAS28 scores fell by 11% from 4.08 (95% CI: 3.91, 4.25) in 2005 to 3.64 (3.34, 3.78) in 2015. A mixed effects maximum likelihood regression model showed the coefficient was − 0.033 (95% CI -0.044, − 0.023) and these falls in DAS28 were highly significant (*p* < 0.001). The reduction in DAS28 scores was predominantly between 2005 and 2010; there were no falls from 2011 onwards. Associated with these falls in mean DAS28 scores was an increase in the number of patients in remission (18% in 2005; 27% in 2015) and the number with active disease (DAS28 > 5.1) decreased (25% in 2005; 16% in 2015).

#### Patients followed over three or more years

There were 4345 annual measures of DAS28 in the 752 patients with at least three annual clinic visits. Their mean DAS28 scores showed similar falls over time (Fig. 1). The mean DAS28 scores fell 12% from 3.95 (95% CI 3.73, 4.17) to 3.48 (95% CI 3.22, 3.74). Trend analysis, which takes into account repeated measures, showed similar changes (Additional file 2: Table S2). The number of patients in remission also increased (20% in 2005; 29% in 2015) and the number with active disease decreased (21% in 2005; 14% in 2015).

### Changes in DAS28 components

#### All patients

**Fig. 2:**
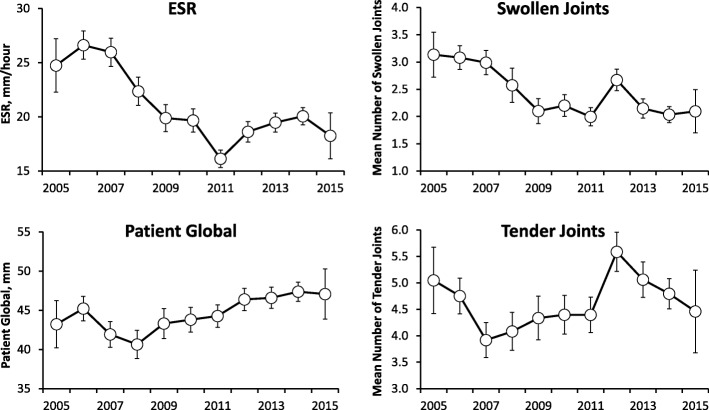
Changes in Components of DAS28 in All Patients (Mean With 95% Confidence Intervals)

Changes in the components of DAS28 showed varied patterns of change over time in all patients (Fig. 2). Swollen joint counts fell by 32% from 3.1 (95% CI 2.7, 3.5) in 2005 to 2.1 (95% CI 1.7, 2.5) in 2015. The ESR showed similar falls of 24% from 25 (95% CI 22, 27) in 2005 to 19 (95% CI 16, 20) in 2005. In contrast tender joint counts fell by only 10% from 5.0 (95% CI 4.4, 5.7) to 4.5 (95% CI 3.7, 5.2) and patient global assessments increased by 9% from 43 (95% CI 40, 46) to 47 (95% CI 44, 50).

#### Patients followed over three or more years

These patients showed a similar pattern of change (results not shown). Swollen joint counts fell by 49%, ESR by 25% and tender joint counts by 17%; but patient global increased by 8%.

### Remission status in patients followed over three or more years

#### Disease activity groups and remission status

Based on the presence of remission the patients were subdivided into 3 sub-groups: 68 (9%) were always in remission, 376 (50%) had one or more episodes of remission without being sustained, and 308 never achieved remission. There were substantial differences between these groups (Table 2). In addition 16% had sustained low disease activity or remission and 58% had point low disease activity or remission at one or more time-points without being sustained.

**Table 2:** Disease Activity Sub-Groups In Patients Followed Over Three Or More Years

	Always Remission	Some Remission	No Remission	Significance
	*(n = 68)*	*(n = 376)*	*(n = 308)*	
Females (%)	34 (50%)	299 (80%)	246 (80%)	*P* < 0.001*
Caucasian Ethnicity (%)	58 (85%)	287 (76%)	195 (63%)	*P* < 0.001*
Black African/Caribbean Ethnicity (%)	1 (2%)	37 (10%)	62 (20%)	*P* < 0.001*
Initial Mean Age In Years (SD)	54 (16)	55 (15)	56 (14)	NS**
Initial Disease Duration In Years (SD)	5 (5)	10 (9)	12 (11)	*P* = 0.003**
Initial Mean DAS28 (SD)	1.65 (0.65)	3.48 (1.32)	4.65 (1.19)	*P* < 0.001**
Overall Mean DAS28 (SD)	1.57 (0.45)	3.06 (0.71)	4.47 (0.82)	*P* < 0.001**
Any Flares (%)	27 (40%)	266 (71%)	194 (63%)	*P* < 0.001*
Overall Mean EQ5D-3 L (SD)	0.77 (0.17)	0.58 (0.24)	0.38 (0.28)	*P* < 0.001**
Initial Mean HAQ (SD)	0.31 (0.50)	1.06 (0.81)	1.58 (0.81)	*P* < 0.001**
Overall Mean HAQ (SD)	0.31 (0.45)	1.03 (0.70)	1.66 (0.67)	*P* < 0.001**
HAQ Below 0.50 On One Or More Occasion (%)	58 (85%)	180 (48%)	56 (18%)	*P* < 0.001*

*Chi-Squared Testing **One Way Analysis Of Variance

In the relatively small group of 68 patients always in remission, there were fewer females (50%), more patients with Caucasian ethnicity (85%) and shorter mean initial disease duration (5 years). The other two groups had similar numbers of females (80%) and similar initial disease durations (10 and 12 years). Patients with intermittent remission included more patients with Caucasian ethnicity (76%) and fewer with Black African/Caribbean Ethnicity (10%) than those without remission (63 and 20% respectively).

By definition overall mean DAS28 scores were lowest in those patients who were always in remission and highest in patients with no remission. Flares (increase in DAS28 ≥ 0.6 between consecutive assessments) occurred in 40% of patients always in remission and 71 and 63% of the other patient groups. Overall mean EQ5D-3 L scores were highest (better) in patients always in remission and lowest in patients with no remission.

Patients who never achieved remission included 14 patients who always had high disease activity (DAS28 > 5.1). All 14 patients were females with longer initial disease durations (17 years); only 8 (57%) had Caucasian ethnicity. They had very low overall mean EQ5D-3 L scores (0.07).

#### Changes in DAS28 scores and remission status

**Fig. 3:**
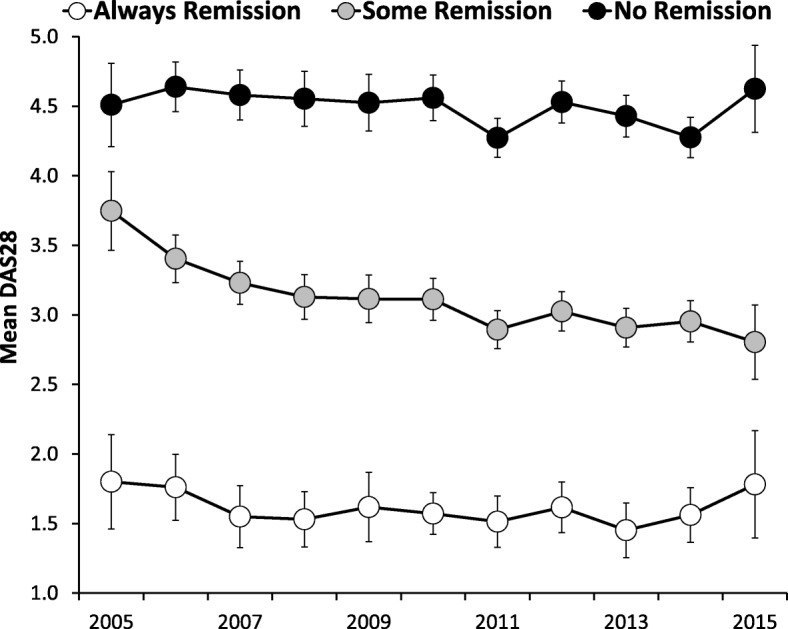
Changes in DAS28 in Three Sub-Groups of Patients Followed Over Three or More Years

The 3 subgroups showed differences in DAS28 scores from 2005 to 2015 (Fig. 3). There were no temporal changes in patients always in remission and patients who never had remission. By contrast in patients with one or more remissions, DAS28 scores fell by 25% from 3.75 (95% CI 3.46, 4.03) to 2.80 (95% CI 2.54, 3.07).

#### Changes in treatment and remission status

**Fig. 4:**
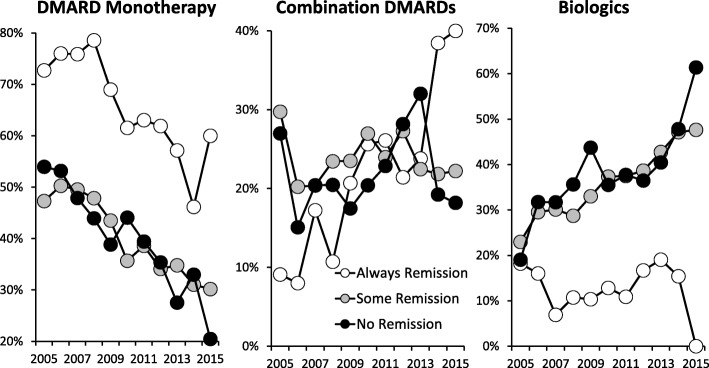
Changes in Treatment Strategies in Three Sub-Groups of Patients in Patients Followed for Three or More Years

The use of DMARD monotherapies, combination DMARDs and biologics changed over time (Fig. 4). Initially 55% of patients were taking DMARD monotherapies and 19% biologics. DMARD monotherapy fell progressively, with increasing use of biologic therapies; by 2015 this had changed to 35% of patients taking DMARD monotherapies and 42% biologics. Similar changes in biologics use were seen in patients followed over three or more years (Additional file 3: Table S3).

Patients always in remission received fewer DMARD monotherapies and biologics compared to patients with some or no remissions. There was strong evidence of increasing intensity of care in line with the intensive treatment strategy; in particular patients with initial moderate disease had increased biologic use rising from under one quarter in 2005 to over one half by 2015 irrespective of remission status (Additional file 4: Figure S2).

#### Disability and remission status

Overall mean HAQ scores were lowest in the patients always in remission. 85% of these patients had HAQ scores below 0.50 on one or more occasions. Overall mean HAQ scores were highest in patients with no remissions and only 18% had HAQ scores below 0.50 on one or more occasions.

**Fig. 5:**
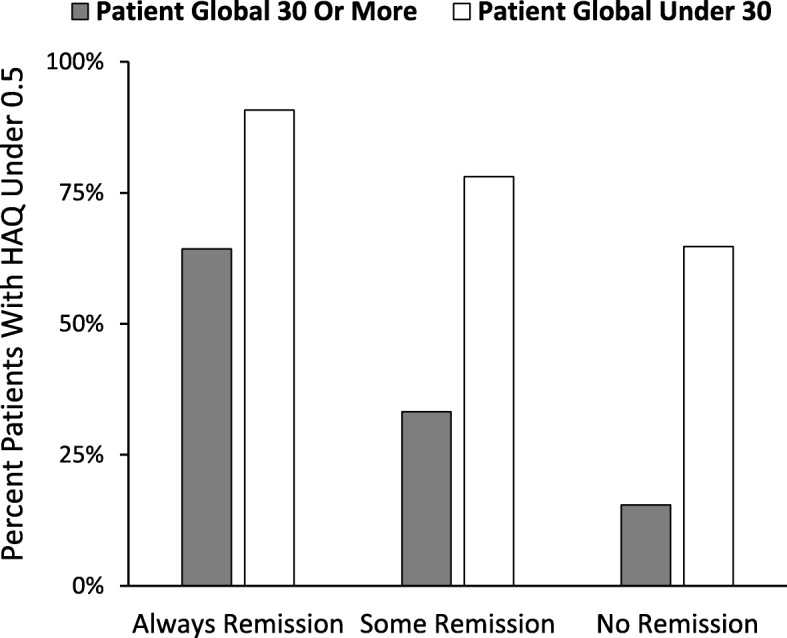
Low HAQ scores are associated with Low Patient Global regardless of remission status

In all three subgroups there was a relationship between low HAQ scores and low patient global scores (Fig. 5). This analysis shows that irrespective of remission status, those patients who had overall mean patient global scores under 30 were significantly more likely to have HAQ scores below 0.5, regardless of their remission status. This difference was significant in all three subgroups (Chi Squared analyses showed that in patients always in remission the significance level of the difference was 0.013; in both other groups it was < 0.001).

Finally in the subgroup with some remission, 62/110 (56%) who had no flares (increase in DAS28 ≥ 0.60) had one or more HAQ scores < 0.5 compared with 118/266 (44%) of patients who had one or more flares. This difference was significant (Chi-Squared with continuity correction 4.02; DF 1; *p* = 0.034). Flares were unrelated to low HAQ scores in the other remission groups.

### Impact of flares

Patients who had more flares had overall higher mean HAQ scores and higher end-point HAQ scores (Table 3). When patients who were always in remission were excluded, the end-point HAQ remained significantly different between remission groups (*P* = 0.043 on one way analysis of variance) but the overall mean HAQ was no longer significantly different.

**Table 3:** Relationship Between Flares And Disability

Number of Flares	Number Of Patients	Overall HAQ *Mean (95% CI)*	Final HAQ *Mean (95% CI)*
0	260	1.08 (0.98, 1.18)	1.19 (1.08, 1.31)
1	290	1.29 (1.20, 1.38)	1.23 (1.13, 1.33)
2	127	1.21 (1.08, 1.35)	1.32 (1.16, 1.48)
3	47	1.50 (1.33, 1.66)	1.53 (1.28, 1.78)
4	14	1.43 (1.11, 1.75)	1.73 (1.27, 2.20)
One Way Analysis Of Variance		*P* = 0.002	*P* = 0.029

### Impact of disease duration

Dividing patients followed for three years or more into those with disease durations of less than 5 years and those with more than 5 years showed no evidence that DAS28 scores were different over time between them (Additional file 5: Figure S1) However, there two differences between them (a) sustained remission was more frequently seen in patients with less than 5 years duration (12% versus 3%; Chi-squared 10.8; DF = 1; *P* = 0.001); (b) flares were more frequently seen in patients with more than 5 years duration (78% versus 62%; Chi-squared 11.9; DF = 1; *P* = 0.001).

## Discussion

Our long-term real world observational study shows both the potential benefits and limitations of a goal directed, intensive treatment strategy for patients with established RA. A preliminary cross sectional analysis of goal directed therapy in these patients compared to a matched group receiving less intensive approaches has previously established the benefit of this approach [25].

During the decade of follow-up reported here, treatment intensities increased, mean disease activity fell and there were more remissions. Patients who achieved one or more remissions had lower levels of disability and higher health related quality of life. However, not all patients responded to intensive treatment and a small minority had persistently active disease with high disability levels and low health related quality of life.

The presence of intermittent and persistent remissions had a substantial relationship with both treatment and disease outcomes. Patients with remissions were more likely to be male and Caucasian with shorter disease duration, which reflects previous experience predicting RA outcomes [32–34]. There were also differences in treatment intensity with patients in persisting remission having less intensive treatment and, in particular, fewer biologics. It is possible patients in sustained remission had any biologic treatment tapered and stopped.

Reduced DAS28 scores over time were only seen in those patients who had intermittent remission. In addition, we found strong relationships between disease activity, disability and health related quality of life. Patients with persistent remissions had the lowest HAQ and highest (best) EQ5D scores and patients who never had a remission had the highest HAQ and lowest EQ5D scores. An extensive body of clinical evidence from observational studies has previously highlighted these relationships, including reports from Alemo et al. [35] and Radner et al. [36, 37]. There is also evidence that with conventional non-intensive treatment, disability increased with disease duration and over time relationships between disease activity and disability change [38]. These findings highlight the importance for patients of controlling active disease. Overall in our patients DAS28 remission frequencies increased from an average of 18% in 2005 to 27% in 2015 in our patients. These remission rates are similar to those reported in trials of biologics in established RA. For example, Kivitz et al. [39] reported 32% remission rates adding tocilizumab to methotrexate and Smolen et al. reported 23% remission rates adding certolizumab to methotrexate in established RA [40]. The DAS28 remission rates were relatively static in a long-term extension study [41].

The decreases in disease activity we observed over time reflect the previous evidence that RA is becoming less severe over time [7–21]. Interestingly we found that some aspects of disease activity, in particular swollen joint counts and the ESR, improved more than others, in particular patient’s global assessments. We also noted patient global assessments were most closely associated with disability scores, reflecting our previous findings in clinical trials [42]. This is of potential clinical importance as it implies need for treatment of aspects of RA beyond inflammatory synovitis to reduce patient global assessments and improve disability in all patient groups. Long-term assessments from the BARFOT study have shown that whilst disease activity has declined over time, disability and pain have not [43]. One explanation for this finding is that some features of RA are not directly driven by synovial inflammation; and may represent “fibromyalgic RA” [44]. Such patients have more pain [45], often fail to achieve remission when treated intensively [46] and have less evidence of active synovitis [47]. It is possible that other drug treatments or non-drug treatments such as psychological support [48] or exercise [49] may be beneficial in these patients. The implication is that intensive drug treatment alone is not sufficient and the use of other approaches to manage established RA needs to be extended. It is also possible there is a lower level of DAS28 which can be achieved by current drug treatments as international comparisons in the QUEST-RA study found no country achieved mean DAS28 scores below 3.0 [50]. In addition the timing of NICE and other guidance may have influenced clinical practice, though we have not found any definite evidence to support this contention. The main strength of our study is that it presents findings from a single centre in which clinical staff followed an agreed management approach for RA based on goal-directed, intensive management approaches and used standardised clinical assessments. It also has several limitations. The principal weakness, inherent in all observational studies, is that we recruited consecutive patients irrespective of their disease severity or activity. It is therefore difficult to be certain how much secular trends in the severity of RA have contributed towards our findings, though recent analysis of early RA patients from England since 1990 does not suggest this has occurred to any great extent [24]. A second limitation is that patients were seen as required rather than in any standardised manner, reflecting current clinical practice. As patients with active disease are likely to be seen more frequently than patients in remission, this approach may over-estimate the relative frequency of active disease, particularly in the final years of the study. A third limitation is that patients were not followed-up if they left the clinic, which is in line with routine clinical practice. Consequently we cannot exclude impacts from censoring or informativeness of the follow-up process. A fourth limitation is that our treatments with biologics were influenced by NICE criteria; greater use of biologics might have improved outcomes even more. A fifth limitation is the definition of flares; other definitions were all developed some time after our study started and are difficult to apply retrospectively. A sixth limitation is that many patients did not remain under follow-up for over three years. The reasons for this are likely to be complex; death is one factor; London has a highly mobile population and patients frequently move within London or outside the capital; RA patients often have comorbid diseases and these can result in their moving to other hospitals for their care; and patients may stop attending specialist units, with historical studies suggest nearly half of RA patients may not be under specialist care receiving DMARDs during the course of their disease [51, 52] Finally, our cohort predominantly included patients with established disease rather than new referrals with early RA. Whilst this most closely reflects routine practice, it means caution must be used when comparing our findings with those from inception cohorts of early RA patients, which will include patients with mild disease who may not require follow up in specialist clinics.

## Conclusions

We conclude intensive management regimens have been associated with progressive improvement in disease activity and more remissions over the last 10–15 years. When patients achieve intermittent or sustained remission their function and quality of life improve. Our treat-to-target, goal-directed therapy strategy, using DAS28 as the measure, was used as a guide to medical therapy, with reductions in swollen joint count and ESR. Although we did not assess longitudinal radiographs in our population, it is likely that improvement in these disease attributes, also noted in other populations, most likely accounts for the substantial reduction in radiographic progression now seen in people with RA [53]. This is a significant achievement, as progressive joint damage was previously common in RA. However, tender joint counts and patient global assessments have improved less and may need alternative or additional treatment approaches. A minority of patients have continued high disease activity with substantial disability and reduced quality of life. Individualised strategies may be required for this group including novel therapies or psychological interventions.

## Additional files

Additional file 1: **Table S1.** Patients seen in each calendar year (DOCX 36 kb)

Additional file 2: **Table S2.** Changes In DAS28 In Patients Followed For Three Or More Years Using Trend Analysis To Take Into Account Repeated Measures (DOCX 43 kb)

Additional file 3: **Table S3.** Treatments Used Over Time (DOCX 56 kb)

Additional file 4: **Figure S2.** Biologic Use In Patients With Initial Moderate Disease Activity And Remission Status (DOC 66 kb)

Additional file 5: **Figure S1.** Changes In Mean DAS28 Over Time In Patients Seen Within Five Years Of RA Onset Or Later (DOC 66 kb)
