# Supplementary material for: Real world long-term impact of intensive treatment on disease activity, disability and health-related quality of life in rheumatoid arthritis
Source: BMC Rheumatol. 2019 Feb 25;3:6. doi: 10.1186/s41927-019-0054-y (PMC6390620; doi:10.1186/s41927-019-0054-y)
Supplement: Supplementary file 1 — Table S1. Patients seen in each calendar year (DOCX 36 kb) [file 41927_2019_54_MOESM1_ESM.docx]

**Supplementary Table 1: Patients seen in each calendar year**

| **Year** | **Patients seen for >3 years (n)** | **Patients seen for <3 years (n)** |
| --- | --- | --- |
| 2005 | 33 | 175 |
| 2006 | 81 | 232 |
| 2007 | 43 | 70 |
| 2008 | 17 | 37 |
| 2009 | 16 | 35 |
| 2010 | 85 | 112 |
| 2011 | 67 | 94 |
| 2012 | 85 | 71 |
| 2013 | 110 | 67 |
| 2014 | 193 | 47 |
| 2015 | 22 | 1 |
