# Supplementary material for: Real world long-term impact of intensive treatment on disease activity, disability and health-related quality of life in rheumatoid arthritis
Source: BMC Rheumatol. 2019 Feb 25;3:6. doi: 10.1186/s41927-019-0054-y (PMC6390620; doi:10.1186/s41927-019-0054-y)
Supplement: Supplementary file 2 — Table S2. Changes In DAS28 In Patients Followed For Three Or More Years Using Trend Analysis To Take Into Account Repeated Measures (DOCX 43 kb) [file 41927_2019_54_MOESM2_ESM.docx]

**Supplementary Table 2.: Changes In DAS28 In Patients Followed For Three Or More Years Using Trend Analysis To Take Into Account Repeated Measures**

| **Year** | **DAS-28** | | |
| --- | --- | --- | --- |
|  | *Mean* | *Lower 95% CI* | *Upper 95% CI* |
| 2006 | 4.17 | 3.97 | 4.36 |
| 2007 | 3.77 | 3.63 | 3.91 |
| 2008 | 3.66 | 3.53 | 3.79 |
| 2009 | 3.64 | 3.50 | 3.77 |
| 2010 | 3.59 | 3.47 | 3.71 |
| 2011 | 3.43 | 3.32 | 3.54 |
| 2012 | 3.59 | 3.48 | 3.70 |
| 2013 | 3.53 | 3.43 | 3.64 |
| 2014 | 3.45 | 3.35 | 3.56 |
| 2015 | 3.30 | 3.10 | 3.51 |
