# Supplementary material for: Real world long-term impact of intensive treatment on disease activity, disability and health-related quality of life in rheumatoid arthritis
Source: BMC Rheumatol. 2019 Feb 25;3:6. doi: 10.1186/s41927-019-0054-y (PMC6390620; doi:10.1186/s41927-019-0054-y)
Supplement: Supplementary file 3 — Table S3. Treatments Used Over Time (DOCX 56 kb) [file 41927_2019_54_MOESM3_ESM.docx]

**Supplementary Table 3: Treatments Used Over Time**

| **Year** | **All Patients** | | | **Patients Followed Over Three Or More Years** | | |
| --- | --- | --- | --- | --- | --- | --- |
|  | *DMARD Monotherapy* | *DMARD Combinations* | *Biologics* | *DMARD Monotherapy* | *DMARD Combinations* | *Biologics* |
| 2005 | 55% | 26% | 19% | 52% | 27% | 21% |
| 2006 | 56% | 16% | 28% | 53% | 17% | 29% |
| 2007 | 53% | 20% | 27% | 51% | 20% | 29% |
| 2008 | 51% | 21% | 29% | 49% | 21% | 30% |
| 2009 | 46% | 21% | 33% | 44% | 21% | 34% |
| 2010 | 44% | 23% | 32% | 41% | 25% | 34% |
| 2011 | 42% | 23% | 35% | 41% | 24% | 35% |
| 2012 | 38% | 27% | 34% | 37% | 27% | 36% |
| 2013 | 37% | 26% | 37% | 34% | 26% | 40% |
| 2014 | 36% | 27% | 37% | 33% | 22% | 45% |
| 2015 | 35% | 23% | 42% | 28% | 21% | 51% |
