# Supplementary figures and images for: Real world long-term impact of intensive treatment on disease activity, disability and health-related quality of life in rheumatoid arthritis
Source: BMC Rheumatol. 2019 Feb 25;3:6. doi: 10.1186/s41927-019-0054-y (PMC6390620; doi:10.1186/s41927-019-0054-y)

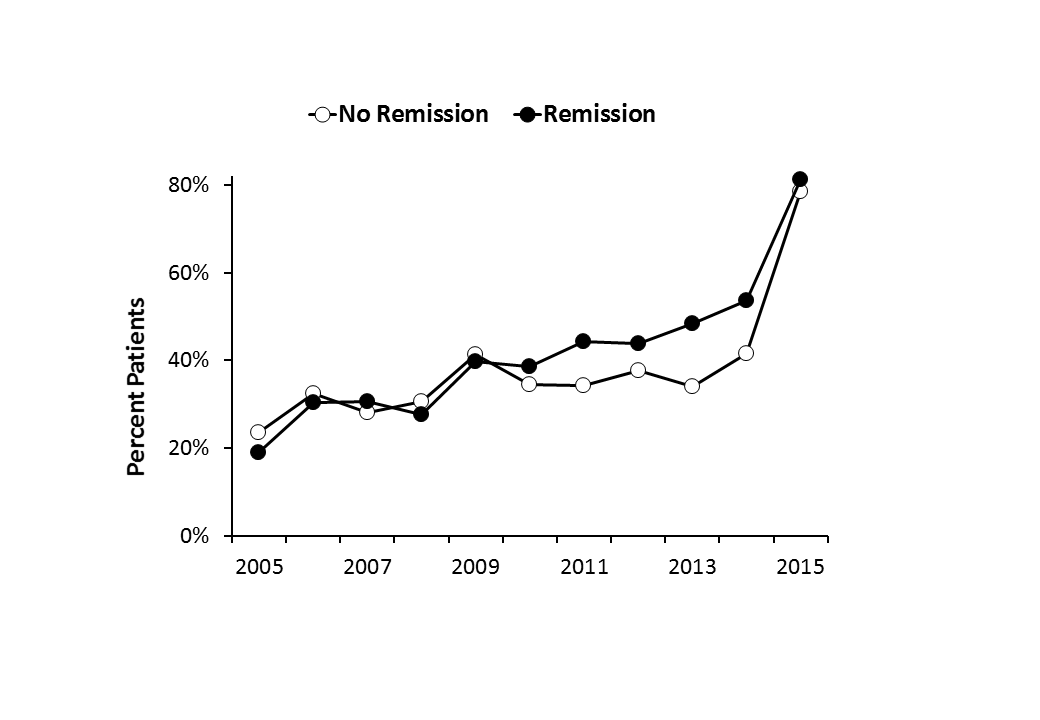

Supplement: Supplementary file 4 — Figure S2. Biologic Use In Patients With Initial Moderate Disease Activity And Remission Status (DOC 66 kb) [file 41927_2019_54_MOESM4_ESM.doc]

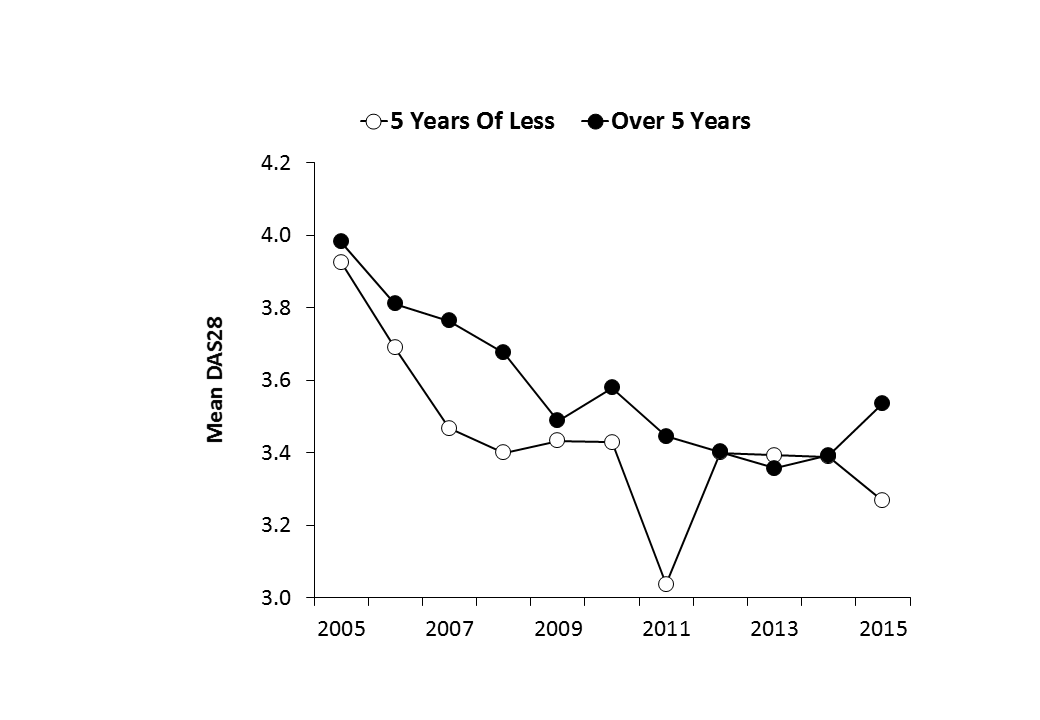

Supplement: Supplementary file 5 — Figure S1. Changes In Mean DAS28 Over Time In Patients Seen Within Five Years Of RA Onset Or Later (DOC 66 kb) [file 41927_2019_54_MOESM5_ESM.doc]
